# Internal friction evidence for the carbides-formation in the high Co–Ni martensite steel during heat treatment

**DOI:** 10.1038/s41598-025-93709-8

**Published:** 2025-03-20

**Authors:** Xian-Yu Li, Gang-Ling Hao, Tao-Li Gao, Wei-Guo Wang, Dan Wang, Zhao-Hui Zhang, Xing-Wang Cheng, Le Wang

**Affiliations:** 1 School of Physics and Electronic Information, Yan’an University, Yan’an, 716000 PR China; 2 School of Materials Science and Engineering, Beijing Institute of Technology, Beijing, 100081 PR China; 3 National Key Laboratory of Science and Technology on Materials under Shock and Impact, Beijing, 100081 PR China; 4 Beijing Institute of Technology Chongqing Innovation Center, Chongqing, 401135 PR China

**Keywords:** Internal friction, M54 steel, Microstructure, M_2_C carbides, Phase transformation, Structural materials, Mechanical engineering

## Abstract

The internal friction (IF) behaviors, combined with X-ray diffraction (XRD), dilatometry, and transmission electron microscopy (TEM) analysis of the cryogenic treated and tempered M54 steel were systematically investigated. In IF-temperature curves, the peak P_1_ was proved to be a Snoek-Ke-Koester (SKK) relaxation peak associated with interstitial carbon atoms in martensite matrix according to its activation energy. The peak P_2_ and P_3_ were attributed to reverse martensite transformation and martensite transformation, respectively, during the thermal cycle. Based on the analysis results of IF, XRD and TEM, M_2_C precipitation indeed occurred during tempering, leading to final ultra-high strength and hardness of the aged M54 steel.

## Introduction

Ferrium^®^M54^®^, as a typical high Co-Ni ultra-high strength steel (UHSS), has attracted much attention due to its excellent Ultimate Tensile Strength (UTS) and Fracture Toughness K1C balance and potential application in aeronautical structures since it was developed by QuesTek Innovations in 2013^1–4^. Obviously, heat treatments (mainly including austenitizing, quenching and cryogenic treatment, and tempering) of high Co-Ni steels can produce a variety of structures with different mechanical properties. Essentially, cryogenic treatment can supply more transformation energy to reduce residual austenite and completely finish martensite transformation^5^. Tempering process is necessary to acquire fine needle-shaped M_2_C precipitates and reverse austenite layers especially for AerMet^®^100 and Ferrium^®^M54^®^^6–9^. A large number of research papers that offer an increasing understanding between microstructures and property changes that evolve as a function of tempering and time have been published. It is pointed by Ayer et al.^10^ that the hardening of the aged AerMet^®^100 steel was mainly attributed to the formation of needle-shaped, coherent precipitates, and the toughening was associated with the absence of coarse cementite particles and the formation of thin-film reverted austenite located at the lath martensite boundaries. Shi et al.^6^ studied the microstructure and mechanical properties of AerMet^®^100 steel tempered at five different temperatures in the temperature range from 472 ^o^C to 492 ^o^C and declared that the mechanical properties were quite sensitive to the tempering temperature. According to A. Mondiere’ research^11^, an excellent strength/fracture toughness balance of Ferrium^®^M54^®^ was achieved with a UTS reaching 1965 MPa and a K1C value up to 110 MPa·m^1/2^ by combination of optimized austenitizing and tempering needed for carbides precipitation.

It is well acknowledged that the efficient and intensive nanometric M_2_C carbides mainly containing Mo and Cr takes place during tempering in the M54 steel, finally leading to its ultra-high strength. Both the cryogenic treatment and aging are kinetic process, during which it is very difficult to in-situ observe the microstructure changes of the M54 steel. To the best of our knowledge, mechanical spectroscopy (internal friction, IF) is the most sensitive detector to the defects and changes in nondestructive materials^12^, which is very helpful to understand the characteristics of motion of internal atoms, inner defects, phase transformation, and relationships between the microstructures and properties^13–16^. Some investigations on low-frequency IF behaviors of the quenched and tempered steels have been reported. Also, some typical IF peaks including Snoek peak, Snoek-Köster peak (related with various occupation or movement behavior of the carbon atoms), and the IF peak associated with phase transformation all have been observed in steels. For instance, the martensitic decomposition behaviors consisting of carbon atom diffusion and carbides precipitation in a low-carbon dual-phase steel were well described by the low-frequency IF method^13^. Li et al.^17^ analyzed the changes of microstructure during the process of tempering transformation for low carbon bainite steel. It was found that the Snoek peak was decreased continuously with increase of tempering time due to decreased interstitial solid solution carbon atoms in bainitic ferrite during tempering process caused by dislocation or carbides diffusion aggregation. Snoek-type IF peak in another low carbon steel, showing the same law as that mentioned above was also detected by Du^18^. It was acknowledged that the Snoek-type relaxation was referred to the stress induced reorientation process of the interstitial atoms in the body-centered cubic (bcc) alloys. That is to say, the migration of interstitial carbon atoms indeed exists and subsequent carbon segregation and carbides formation occur during long aging process. Therefore, the matrix is depleted from carbon after tempering. Carbon segregation during low temperature tempering in a medium carbon steel was also found by Y. Xiao^19^ using thermoelectric power (TEP) method. So far as we know, mechanical spectroscopy investigations on high Co-Ni ultra-high strength steel (UHSS) have not been reported. With the aim of better understanding microstructural evolution of the Ferrium^®^M54^®^ steel during heat treatment, the IF behaviors are systemically investigated for cryogenic treated and tempered Ferrium^®^M54^®^ martensitic steel in this paper.

## Experimental materials and methods

**Fig. 1:**
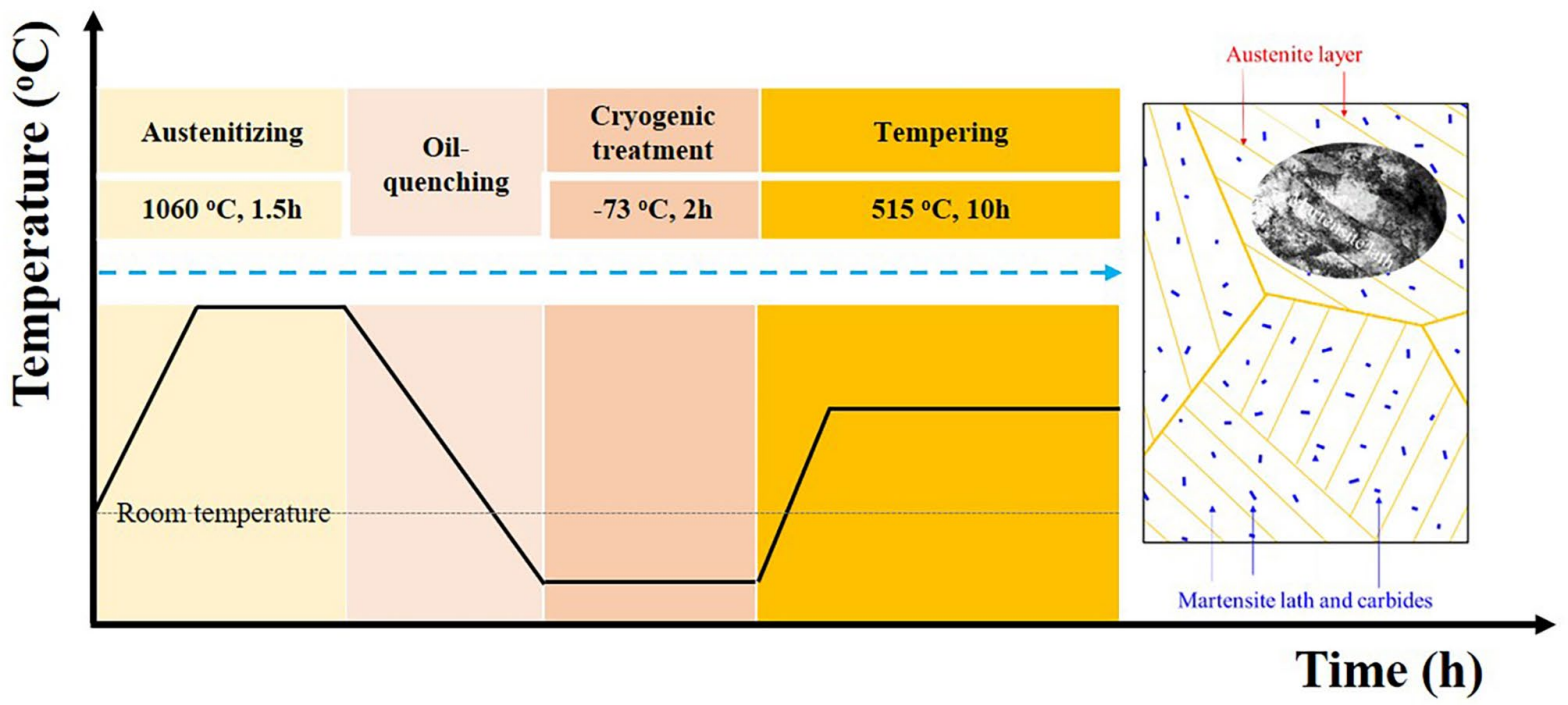
The sketch map of the whole heat treatment process.

The as-received Ferrium^®^M54^®^ steel produced by vacuum arc remelting after vacuum induction melting was supplied as a forged cylinder, 170 mm in diameter. The main chemical compositions of the steel are listed in Table 1. The internal friction specimens with dimension of 65 × 2 × 1 mm^3^ were cut from the steel bar at 1/2 radius along the axial direction using WEDM machine. All the specimens underwent standard heat treatment process, which can be obtained in Ref^20^. The whole heat treatment process is well illustrated in Fig. 1. The specimens firstly austenitized at 1060 ^o^C for 1.5 h, followed by oil quenching to room temperature, and then immediately transferred into a mixture of dry ice and alcohol (-73 °C) for 2 h and subsequently spontaneously heated to room temperature in air. After cryogenic treatment, the specimens were reheated to 515 °C for 10 h and air-quenched to room temperature. Hereafter this whole heat treatment process, the tempered specimens were obtained.

**Table 1:** Main compositions of the tested M54 steel (wt%).

C	Co	Ni	Cr	Mo	V	W	Fe
0.3	6.9	10.3	1.03	1.82	0.08	1.08	Balance

The internal friction (IF, ^−1^) and relative dynamic modulus (RDM) of the cryogenic treated and tempered specimens were measured using a multi-function internal friction apparatus. The IF apparatus was mainly consisted of an inverted torsion pendulum, a photoelectric converter, a resistance heating furnace control system based on PID control, and a computer-controlled system to collect data and operate the whole experimental procedure. The basic principle and relevant parameters of the IF apparatus can be referred to treatise^21^. The IF measurement was carried out using forced-vibration mode in the temperature range from room temperature (RT) to 800 ^o^C in a vacuum atmosphere. The strain amplitude of 2 × 10^−5^ and measurement frequencies of 0.5 Hz, 1.0 Hz, 2.0 Hz and 4.0 Hz were employed to ensure the sample can vibrate and investigate the relationship between IF and frequencies.

Dilatometry experiment was performed using a Netzsch apparatus, DIL402C to accurately determine phase transformation temperature of the cryogenic treated M54 steel. Specimens in the form of a cylinder of diameter 6 mm with a length of 25 mm for dilatometry test were adopted. Samples were heated at 3 ^o^C/min and cooled in the furnace under argon atmosphere. XRD characterizations of the cryogenic treated and tempered sample were carried out using a Panalytical X’Pert PRO diffractometer equipped respectively with a Cu or Co radiation source. The TEM observations were carried out in a JEOL JEM-2100 F operated at 200 kV. TEM foils were prepared by mechanically polishing samples to a 150 μm thickness, and subsequently electro-polishing the thin foils at room temperature.

## Experimental results and discussions

### Experimental results

**Fig. 2:**
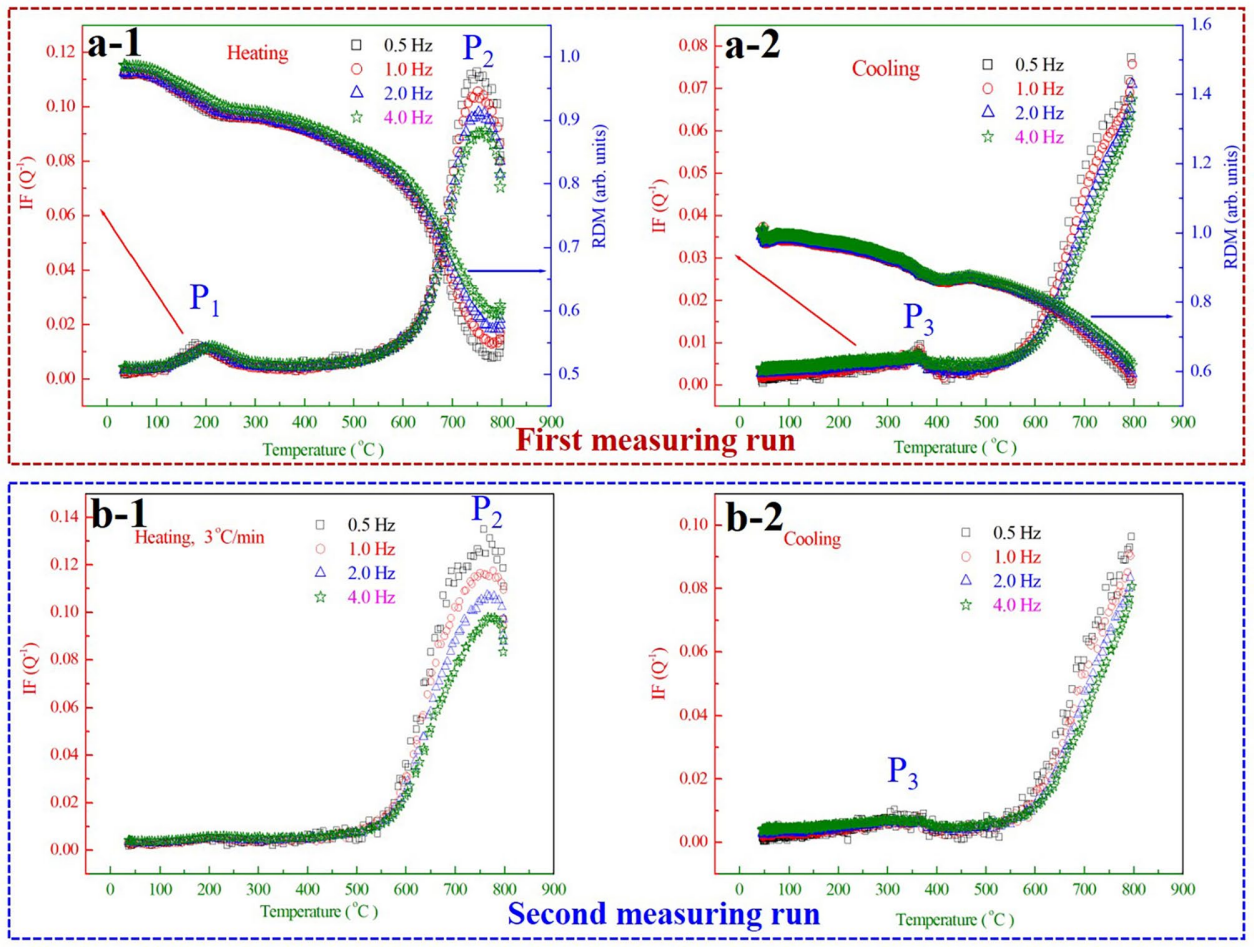
Temperature dependence of IF and RDM of cryogenic treated M54 steel for (a) the first measuring run and (b) the second measuring run.

Figure 2 shows the typical internal friction (IF) and relative dynamic modulus (RDM) spectrums of the deep cryogenic treated M54 steel measured at different frequencies (0.5 Hz, 1.0 Hz, 2.0 Hz and 4.0 Hz) in the temperature range from room temperature to 800 ^o^C. For the first measuring run, shown in Fig. 2(a), the most outstanding features of the IF curves in the spectrum are the appearance of two IF peaks as marked by the arrows with different colors existing at around 200 ^o^C (designated _1_) and 750 ^o^C (designated _2_) corresponding to the heating process. Correspondingly, the RDM exhibits an obvious softening effect when the IF peaks appear, indicating internal changes in its microstructure of the cryogenic treated M54 steel. In addition, the peak *P*_1_ is sensitive to the measuring frequency, the peak position of which shifts towards a higher temperature when the vibration frequency increases, revealing a thermally activated relaxation mechanism. Differently, the position of the peak *P*_2_ is independent of the measuring frequencies and the peak height is inversely proportional to changing frequencies, showing no relaxation characteristics. Also, the corresponding RDM with a local minimum at the peak *P*_2_ characterizes the nature of phase transition. Both the peak *P*_1_ and *P*_2_ are no longer observed and only one small IF peak (termed *P*_3_) appears at about 350 ^o^C in the subsequent cooling, which has similar tendencies with the peak *P*_2_. That is to say, the peak *P*_3_ is also probably attributed to phase transformation process of the cryogenic treated M54 steel during the cooling.

In the subsequent repeating heating process for the same cryogenic treated specimen, shown in Fig. 2(b), the original peak *P*_1_ also disappears, but the peak *P*_2_ reappears at the same temperature of 750 ^o^C. The peak *P*_3_ also appears again in the second cooling process. According to the above experimental results, it is reflected that the peak *P*_1_ is thermodynamically irreversible and possibly related to atomic migration during the heating. The peak *P*_2_ at about 750 ^o^C only appears in the heating process, on the contrary, the peak *P*_3_ only appears in the cooling measurement. So, it is conjectured that a certain inherent relation exists between the peak *P*_2_ and *P*_3_, most likely to be accompanied by martensitic transformation and the reverse transformation process, a common phenomenon in martensite steel during the thermal cycle.

### Discussions

**Fig. 3:**
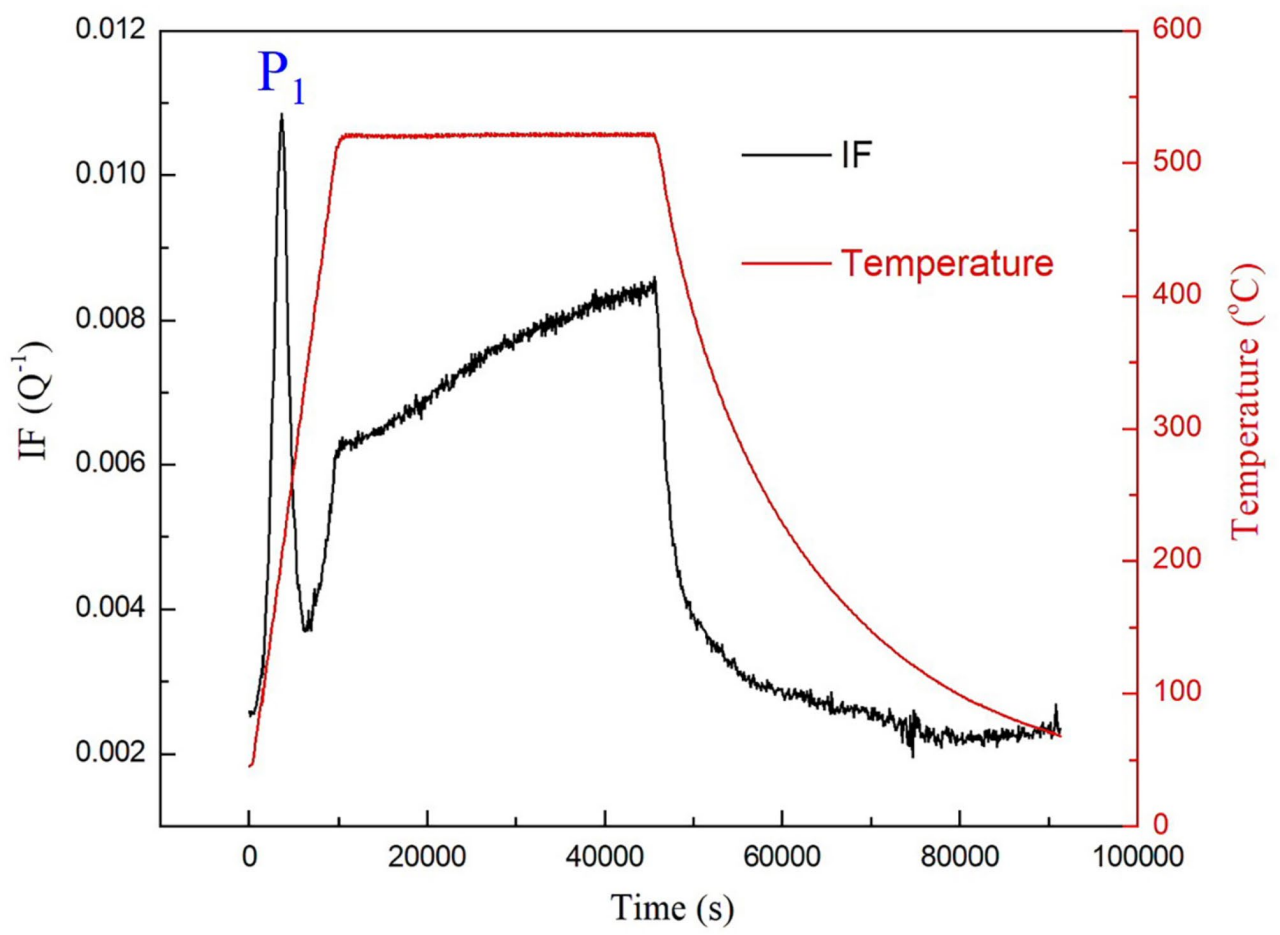
The IF as a function of time for the cryogenic treated M54 steel measured at 2 Hz.

Aiming to prove the above hypothesis and clarify the corresponding IF mechanism of the three IF peaks (*P*_1_, *P*_2_ and *P*_3_), the IF as a function of time for the deep cryogenic treated specimen was investigated in the temperature range from RT to 520 ^o^C and soaking time for 10 h, at a heating rate of 3 ^o^C/min, as shown in Fig. 3. With the change in temperature, the IF curves can be divided into three stages: heating process, soaking-stage and cooling process. It can be clearly found that only the peak *P*_1_ appears in the heating process. In the soaking stage of 10 h, the IF level gradually increases with the increase of time due to improvement of atomic thermal motion. In the cooling process, the IF gradually decreases and the IF peak *P*_3_ is not observed again. It is mentioned in the Ref^7,11^. that mainly dispersed needle-like M_2_C carbides and reverted austenite film along the boundaries of lath martensite both formed during the aging at 515 ^o^C for 10 h. That is to say, the two phenomena can hardly be in situ detected using the IF measurements though it is quite sensitive to the defects and microstructure changes in material. So, it is can be concluded that the appearance of the peak *P*_1_ is not directly caused by precipitation of carbides. And, the appearance of peak *P*_3_ is dependent on the temperature of peak *P*_2_ occurring. That is, both the P_2_ and P_3_ occur or disappear at the same time during the heating and cooling respectively. So, it is inferred that the peak *P*_3_ appears only when the heating temperature exceeds the temperature of peak *P*_2_. It means that phase transformation happened when the temperature reached the temperature of peak *P*_2_, the newly formed phase then subsequently transferred in into origin phase during the cooling. Therefore, the appearance of the *P*_2_ and *P*_3_ is most probably associated with phase transition process (reverse martensitic transformation and martensitic transformation) of the steel during the heating and cooling.

**Fig. 4:**
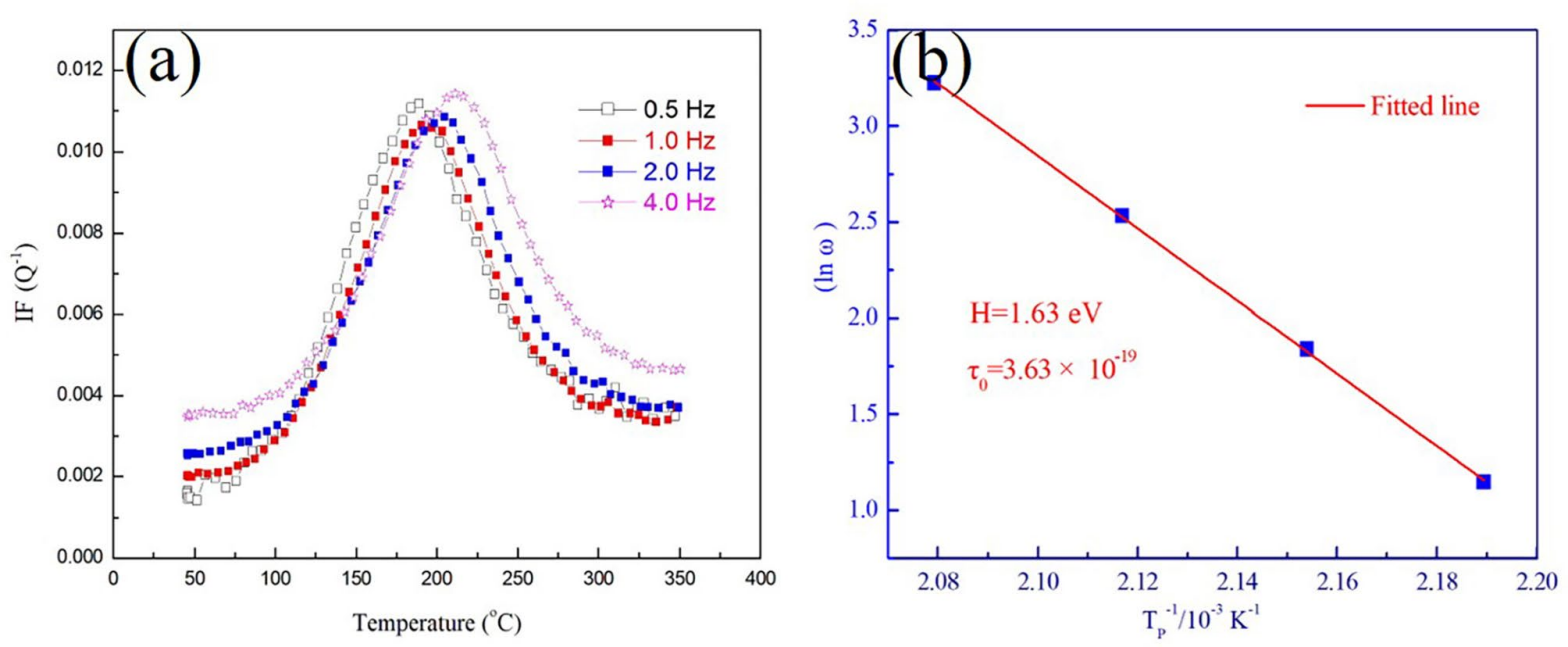
Relaxation characteristics of the *P*_1_ and (b) fitted Arrhenius relationship in the tempered M54 steel sample.

As a relaxational-type IF peak *P*_1_, its activation energy of the relaxation process can be regarded as an important parameter to identify its mechanism. The theoretical equations for the Snoek relaxations can be derived from the general theory of point-defect relaxations for the special case of tetragonal defects in cubic crystals as described in the treatises of Nowick, Berry and Weller^8,11^. The activation energies of this IF peak can be calculated according to formula by its exact peak temperature and frequency. The extra peak temperature of the *P*_1_ can be attained from the IF-temperature curves by deducting background internal friction. Also, the relaxation time *τ* of thermally activated relaxation process should follow the Arrhenius relation:

1 $$\:\tau\:={\tau\:}_{0}\text{exp}(H/{k}_{B}T)\:\:\:$$

where *τ* is the relaxation time, *τ*_0_ is the pre-exponential factor, and *H* is the activation energy, *k*_B_ is the Boltzmann factor and *T* is absolute temperature. The following formula:

2 $$\:\text{ln}\left(2pf\right)=-ln{\tau\:}_{0}-H/{k}_{B}{T}_{p}$$

can be derived under the given condition equation at the peak temperature:

3 $$\:w{\tau\:}_{p}=1$$

where ω is angular frequency and $\:\omega\:=2\pi\:f$, $\:f$ is vibration frequency, and $\:{\tau\:}_{p}$ is relaxation time at peak temperature. Hence, according to the Fig. 4(a) and (b), the *H* and $\:{\tau\:}_{0}$ on the heating curves can be calculated to be *H* = 1.63 eV and $\:{\tau\:}_{0}$= 3.63 × 10^− 19^ s.

**Fig. 5:**
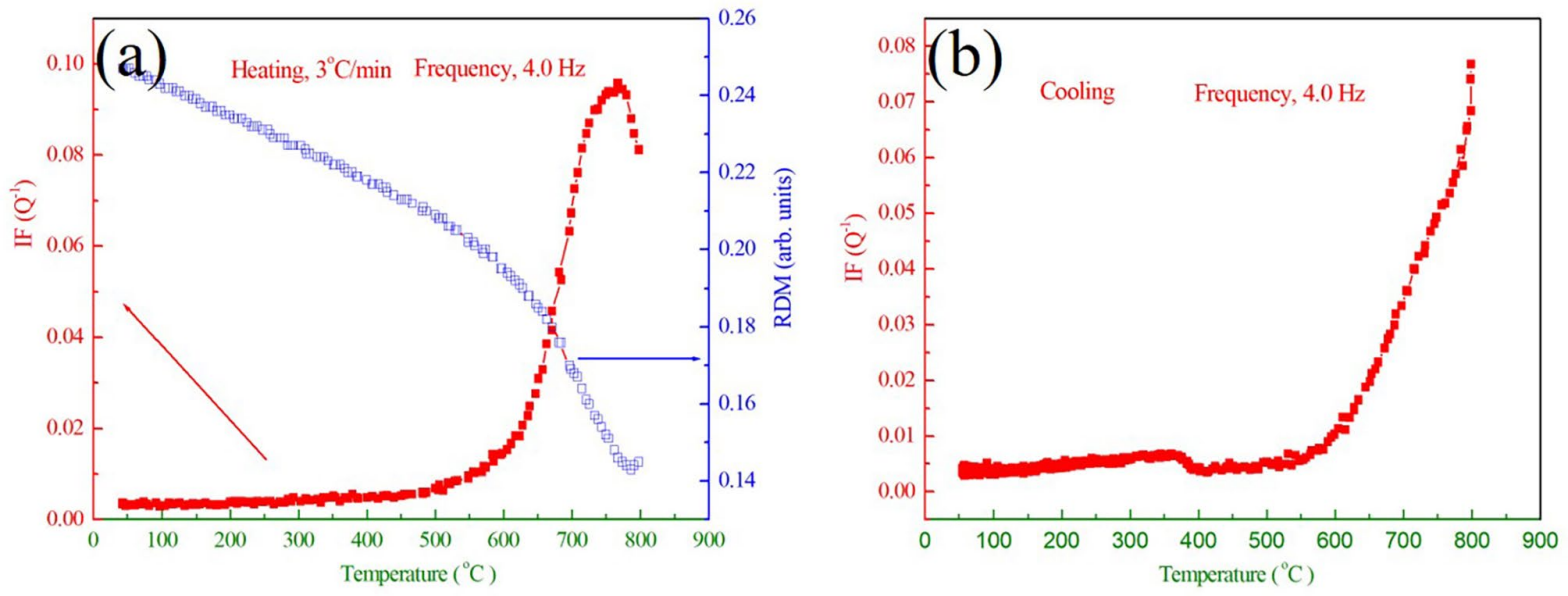
The IF spectra of tempered specimen during the (a) first heating and (b) cooling process measured at 4 Hz.

Based on the IF behaviors and calculation results, it is believed that the Peak *P*_1_ is a Snoek-Ke-Koester (SKK) peak caused by migration of interstitial carbon atoms in martensite matrix because of the similarities of the activation energy (*H* = 1.63 eV) obtained in measurements made for carbon diffusion in α-Fe. There is no doubt that the peak *P*_1_ is associate with the concentration of *C* interstitial atoms, the height of which should be proportional to the carbon atoms in martensite matrix. It can be widely accepted that the interstitial carbon atoms were significantly decreased after tempering because of carbides precipitation during the tempering process. Thus, if the deep cryogenic treated specimen is suffered to tempering treatment, the *P*_1_ would not appear due to consumption of the C atoms in the tempering process during the heating measurement. As expected, on the IF curves of the tempered specimen, only the IF peak *P*_2_ during the heating and the IF peak *P*_3_ during the cooling appeared, as shown in Fig. 5. The IF experiment results of the tempered specimen in the measuring cycle further revealed that the IF peak *P*_1_ is indeed caused by the migration of the interstitial carbon atoms.

**Fig. 6:**
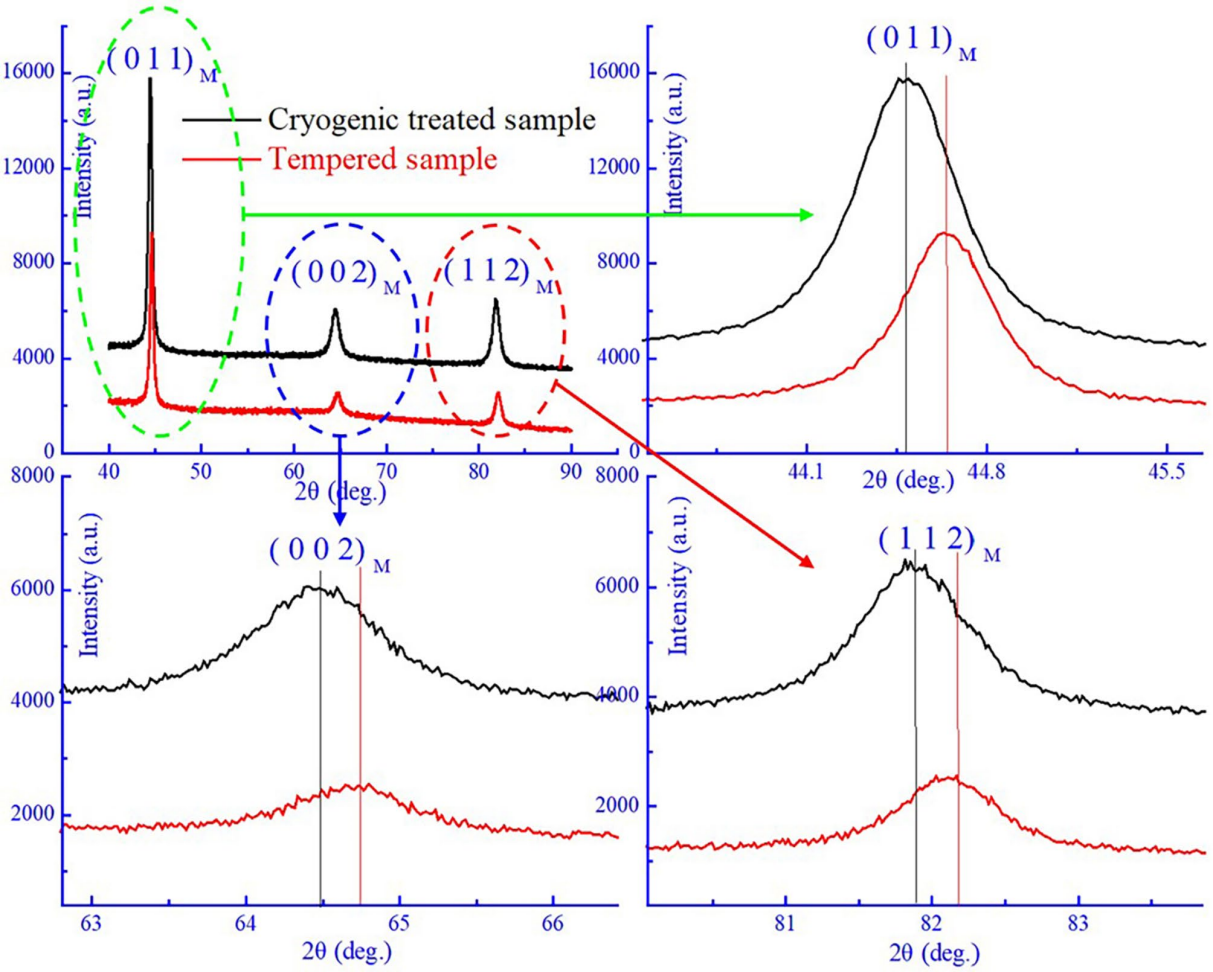
XRD profiles of the cryogenic treated and tempered sample and peak movement of martensite.

XRD, as an effective analysis method, can be usually used to analyze crystal structure evolution of material. Figure 6 shows the XRD profiles of the M54 steel with different states. It is clearly that only (0 1 1), (0 0 2) and (1 1 2) crystal planes of martensite in both the steels were observed. The peak of austenite or carbides could not be observed due to their extremely low content and small size exceeding the test limit of XRD. The above results demonstrate that the structure of the both steels with different state is mainly the martensite. In addition, it is worth noting that the peak of (0 1 1)_M_ crystal face diffraction in martensite is 44.50^o^ and 44.65^o^ respectively for the cryogenic treated and tempered sample, shown in larger images marked with green ellipses in Fig. 6. The peak of (0 1 1)_M_ in tempered sample was about 0.15^o^ larger than that in cryogenic treated sample. Meanwhile, the peak movement of (0 0 2)_M_ and (1 1 2)_M_ also can be found for the two samples with different states in another two larger images in Fig. 6, indicating that the carbon content in the martensite matrix was decreased due to consumption by the formation of carbides during tempering process. That is also to say, *C* consumption leads to a significant decrease of the Mo content in the martensite matrix of the tempered sample because the main type of carbides in this steel is M_2_C with rich Molybdenum. Thus, a larger interplanar spacing for the cryogenic treated sample is obtained, which causes the XRD peaks to shift toward lower diffraction angles according to the well-known Bragg equation. This XRD results could also prove that the migration of *C* atoms first take place and then formation of carbides was subsequently occurred by segregation to dislocations during tempering process in the M54 steel. The same phenomenon that a major drop in interstitial carbon content was caused by tempering was also observed using the thermoelectric power (TEP) method^19^.

**Table 2:** Compressive mechanical properties and Brinell hardness of the cryogenic treated and tempered sample.

Sample	*R*_mc_ (MPa)	HBW5/750
Cryogenic treated sample	2037	594
Tempered sample	2299	566

The mechanical properties of the steel with different state were also listed in the Table 2. It can be found that the compressive strength (*R*mc) of the tempered sample is higher than that of the cryogenic treated sample, but, the surface Brinell hardness (HBW5/750) under a load of 750 kgf is lower. The results were well in accord with the investigation mentioned in introduction. It can be illustrated that martensite decomposition and refinement, carbides precipitation took place during the tempering, leading to an increase of the strength. Additionally, reverted austenite belonging to soft phase formed during the tempering, which leading to a decrease of the hardness. The decrease of hardness suggests that the hardness was not only associated with dislocation density due to the influence of interstitial carbon content in martensite but also influenced by the presence the other phases.

**Fig. 7:**
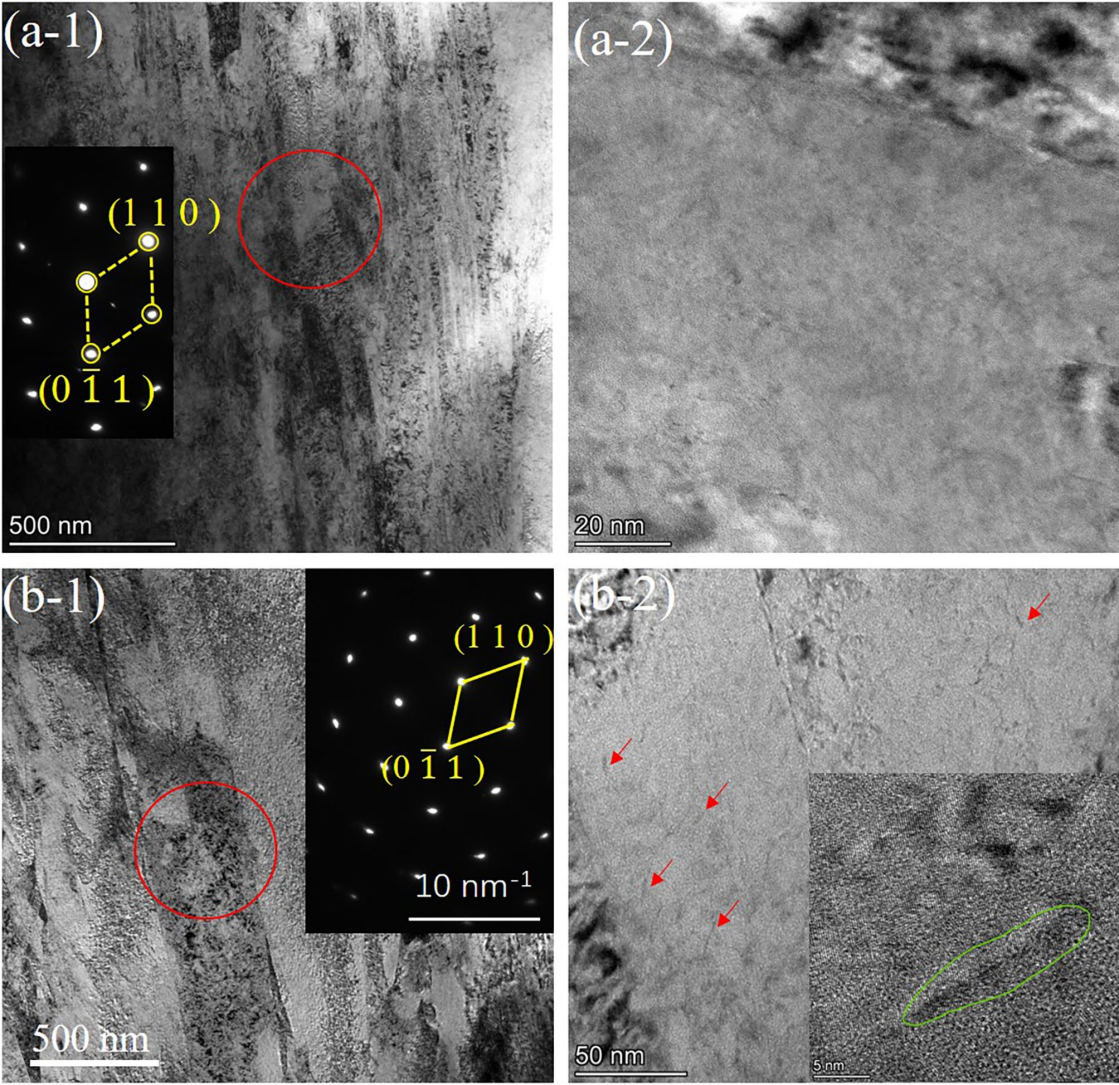
TEM observation of martensite and diffraction pattern on the (a) cryogenic treated sample and (b) aged sample.

Figure 7 shows the morphology images of martensite on the cryogenic treated and tempered specimens. It is obvious that only lath martensite was observed and carbides was hardly found in the martensite matrix of the cryogenic treated specimen shown in Fig. 7(a), suggesting that the carbides precipitating did not happen in the oil quenching and cryogenic treatment process. Differently, the martensite lath could be easily observed and the width of them was not uniform (from 50 to 250 nm) in the tempered specimen, as shown in Fig. 7(b). Besides, the precipitated carbides in martensite were also observed as shown in the illustration in Fig. 7(b). The needle-like carbides present in the steel were charactered by very small size (about 10 nm length). And, some of the precipitations were only element rich zone instead of forming the carbides with a stable crystal structure. Similar needle-like carbides have been reported in previous research, and some relevant experiment results reveal that these carbides which are believed to be rich-Mo M_2_C play a crucial role in improving strength of the steel due to forming the strong interfacial adhesion with the martensite matrix. Thus, the fine-scale carbides could inhibit inclusion induced failure during loading process and improve the strength of the steel. The results of TEM observation further certify that the carbides precipitation does occur in the aging process, resulting in a decrease of the C and Mo content in the martensite matrix.

**Fig. 8:**
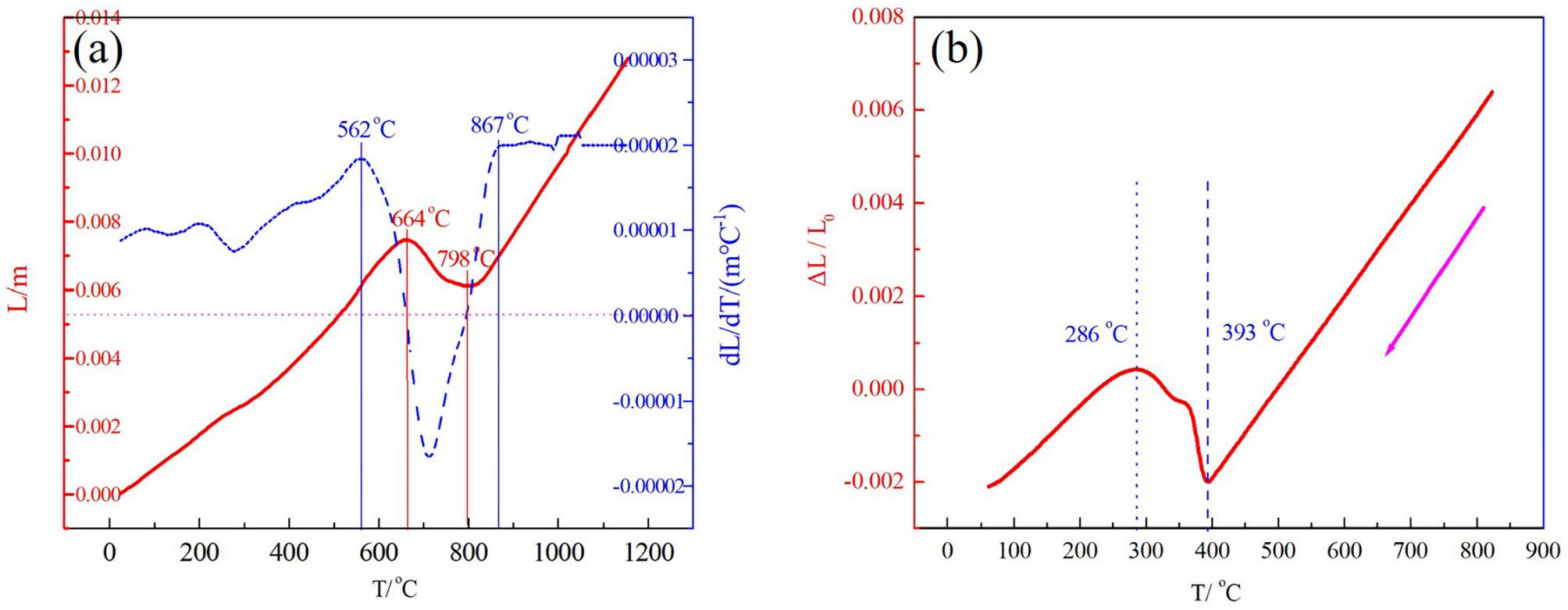
Dilatometer curve for cryogenic treated specimen in a heating rate 3 ^o^C/min.

The phase transformation temperature can be accurately determined by thermal expansion tests. Aiming to detect the Ac1 and Ac3 of the M54 steel, the thermal expansion test was conducted in temperature range from room temperature (RT) to 1000 ^o^C at heating rate of 3 ^o^C/min, shown in Fig. 8(a). It can be found that the Ac1 and Ac3 were 562 ^o^C and 867 ^o^C, respectively. In order to repeat thermal cycle of the IF measurement and determine martensite start transformation temperature (M_s_) and martensite finish transformation temperature (M_f_), another thermal expansion test was conducted in temperature range from RT to 800 ^o^C, followed by furnace cooling, shown in Fig. 8(b). It is clearly that the M_s_ is about 393 ^o^C and the martensite finish transformation temperature is about 286 ^o^C. It was reported that the M_f_ temperature of the tempered steel was lower than that in room temperature. The phase temperature is not only associated with state of the specimen but also heating or cooling rate. Here, the higher M_s_ temperature and M_f_ temperature is probably caused by the slow cooling process and the cryogenic treated specimen. It is obvious that the austenite-transition occurs during 562 ~ 867 ^o^C and martensite-transition occurs during 286 ~ 393 ^o^C. The IF peak P_2_ and P_3_ appear at about 750 ^o^C and 350 ^o^C respectively, which are all well corresponding to the martensitic reverse transformation and martensitic transformation in the steel during thermal cycle.

**Fig. 9:**
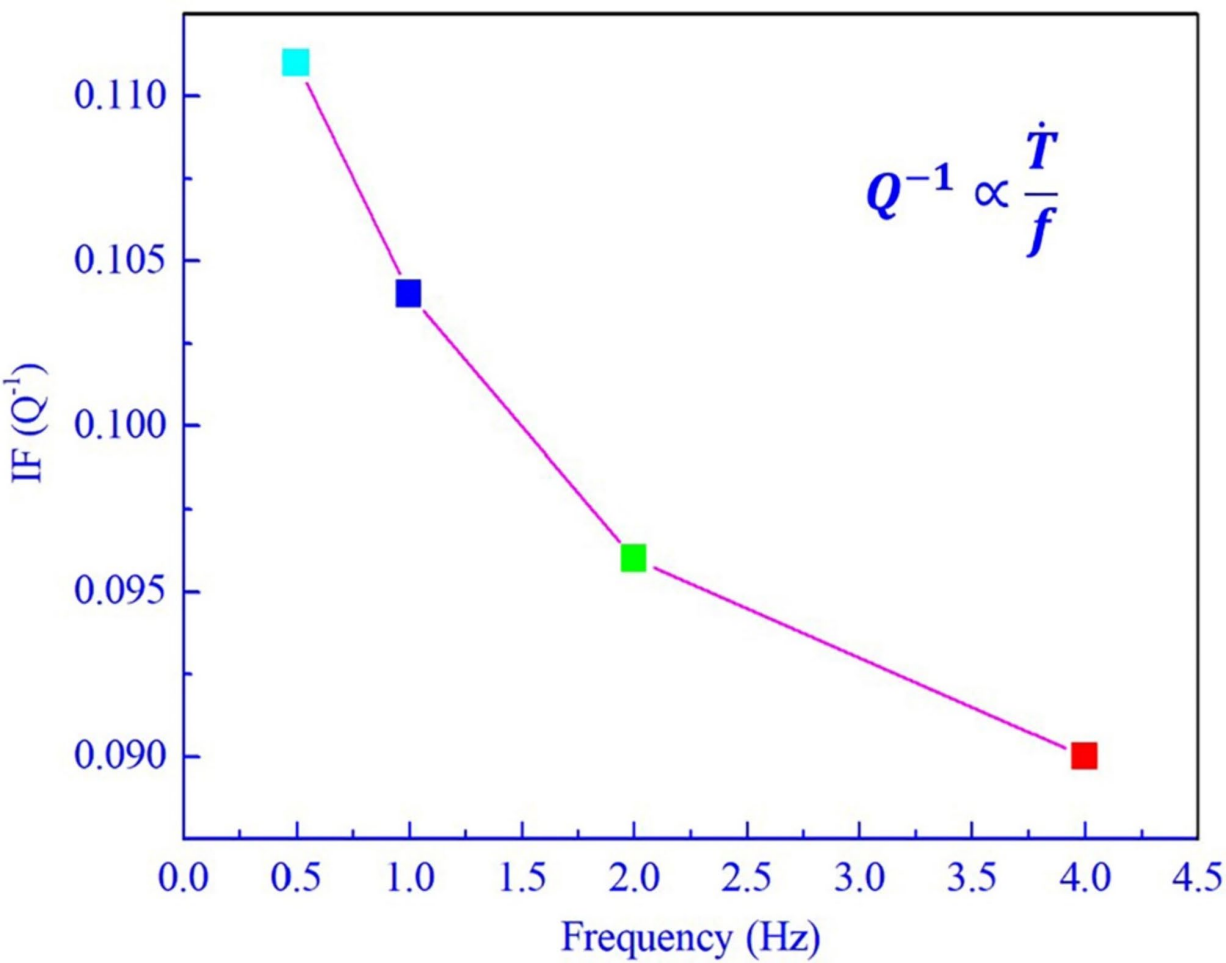
The relationship between the IF (Q^-1^) of the P_2_ and the value of the $\:\frac{\dot{T}}{f}$ .

The net peak height of the P_2_ can also be attained by deducing background IF. It has been proved that the internal friction (IF or *Q*^− 1^) of phase transformation satisfies the following equation:

4 $$\:{Q}^{-1}\propto\:\frac{\dot{T}}{f}$$

where $\:\dot{T}$ is heating rate, f is measuring frequencies. According to the fitting result, the *Q*^-1^ of the P_2_ is linearly proportional to the $\:\frac{\dot{T}}{f}$, shown in Fig. 9, illustrating that the IF is well conformed to the Delorme phase transformation model. It is can be concluded that the appearance of this IF peak P_2_ was definitely originated from martensitic reverse transformation during heating. Here, the same analysis and theoretical derivation on the peak P_3_ will not be repeated owing to the similarity to the peak P_2_. Therefore, it is can be concluded that the peak P_2_ and P_3_ originated from martensitic transformation and reverse transformation in the steel during thermal cycle.

## Conclusion

The IF technology, a dynamic nondestructive detecting method could provide atomic-scale information during the temperature changing process, has exhibited a unique advantage in characterization of structural evolution and phase transition related to migration of carbon atoms. This investigation show that it has been developed as an effective analytical method for further understanding the structural evolutions associated with carbon atoms movements in high Co-Ni martensitic steel by analyzing the IF physical mechanisms.

In this study, the low-frequency mechanical spectroscopy of the M54 steel with different states was investigated by forced vibration mode combining XRD, thermal expansion, and TEM analysis. From the variation of IF-temperature curves, there exist three IF peaks: P_1_ at 200 ^o^C, P_2_ at 700 ^o^C and P_3_ at 350 ^o^C, respectively. Peak P_1_ is a SKK peak attributed to the migration of interstitial carbon atoms in martensite matrix. Peak P_2_ and P_3_ are originated from martensitic reverse transformation and martensitic transformation in the steel during thermal cycle. The XRD diffraction shows that the peak of M (0 1 1) in tempered sample shifts towards larger angle, indicating that the carbon content in the martensite matrix decreased due to consumption by the formation of carbides during tempering. Martensitic reverse transformation and Martensitic reverse transformation temperature in the steel during the same thermal cycle accurately determined by thermal expansion tests are all well corresponding to temperature of the IF peak P_2_ and P_3_. TEM observation further clearly demonstrates that M_2_C carbides indeed existed in tempered specimen.

## Data Availability

The datasets used and analyzed during the current investigation are available from the corresponding author upon a reasonable request.
